# Dangerous and Fatal Results from the Use of Hydrate of Chloral

**Published:** 1871-09

**Authors:** H. W. Fuller

**Affiliations:** Senior Physician to St. George’s Hospital


### Dangerous and Fatal Results from the use of Hydrate of Chloral.

By H. W. Fuller, M.D., F.R.C.P., Senior Physician to St. George's Hospital

As the hydrate of chloral is being largely administered as a sedative and hypnotic, and is often recklessly employed in excessive doses, as if it possessed no noxious properties, it may be well to record a few cases in which moderate doses were productive of dangerous and even fatal results.

On the 9th of February, 1870, J. S——— was admitted under my care into St. George's Hospital, suffering from slight anasarca and bronchitis, connected with Bright's disease. He was restless and nervous, and unable to sleep, and therefore, after he had been some days in the hospital, and was exhausted for want of sleep, I ordered the chloral draft of the hospital (containing thirty grains of chloral) to be taken at bedtime. Soon after he had taken it, he jumped up in bed, clutched at his heart, and complained that the medicine produced a sense of burning. In the course of a few minutes he became violently delirious, and though, after a time, the delirium subsided, so much depression ensued that Dr. Jones, our resident officer, had great difficulty in sustaining his heart's action. Gradually, however, the heart recovered itself, the pulse returned at the wrist, and in a few hours he was out of danger.

Having just read M. Liebreich's assertion that hydrate of chloral, when in contact with an alkali, is transformed into chloroform and formic acid, it occurred to me that the extraordinary results, witnessed in my patient's case, might be attributable to an alkaline condition of the stomach, whereby the chloral was at once converted into chloroform, and thus induced the symptoms which were observed. I therefore determined to try it once again, taking care, on this occasion, to guard against such an occurrance, by administering the chloral in combination with a full dose of acid. The result, however, was precisely the same as on the first occasion. Again there was the same sense of burning and oppression at the chest, followed first by violent excitement and delirium, and subsequently by collapse, with failure of the heart's action; and on this occasion, as on the last, Dr. Jones was long doubtful whether the man would recover. I need not add that I did not make trial of a third dose, even experimentally.

From that time until the first day of the present year I did not meet with any case which led me to question the harmlessness of chloral. I had given it to hundreds of patients, in doses varying from ten grains up to forty-five grains, and had been called in consultation to two patients, one of whom had harmlessly taken two drachms and a half, and the other three drachms, in the night prior to my visit. In some instances it failed as a hypnotic ; in some it produced headache; and in others it gave rise to more or less excitement; but in none was it followed by any symptoms calculated to cause alarm.

On the first of last January, however, I was called in consultation, to see a case in which thirty grains of the hydrate of chloral proved fatal. The patient, a young lady, aged twenty, who was previously in fair health, complained, on the 29th of December, of constipation, and other symptoms of stomach derangement, for which her medical attendant administered a pill at night, followed by an aperient draught in the morning. On the 30th the bowels acted, and she was relieved; but she passed a restless night, and, on the 31st, complained of uneasiness in the lower part of the abdomen, which was attributed to the approaching menstrual nisus. As she was very hysterical, a neighboring practitioner was sent for early, and when he met the family medical attendant in the afternoon, they determined, as she was nervous and restless, and had obtained little or no sleep on the previous night, to give her thirty grains of chloral. She took the dose about 10 p.m. on the 31st, and almost immediately became much excited, and complained of pain in the chest. In about an hour the excitement passed off, and she fell asleep, and slept heavily all night. In the morning she was sleeping so heavily, and looked so pale, that the family became alarmed, and sent for the gentleman who had seen her the previous day. When he arrived, she was very pale, and breathing heavily—a sort of deep sighing respiration; there was no pulse at the wrist, and her extremities were rather cold. It was impossible to rouse her in the slightest degree. He gave her stimulants, and applied warmth to the extremities, and gradually the pulse returned to the wrist, though, at the best, it was only just perceptible. The family medical attendant subsequently met him in consultation, and together they did all that appeared to be expedient; but as everything failed to rouse her, or in any way to alter her condition, they asked me to meet them in consultation at 2 p.m.

When I saw the patient, she was lying on her back, with her eyes closed, and breathing heavily, the respiration having a distinctly sighing character. She was very pale, and somewhat cold; the skin was dry; the pupils were large and dilated, but acted sluggishly under the influence of strong light; the pulse was scarcely perceptible, but the heart was beating regularly, about 120 in a minute, and though its action was very feeble, its sounds were clear and its rhythm was normal. There was no distention of the abdomen—indeed, it was flat and soft; there was no contraction, or rigidity, or undue flaccidity of the limbs. It was impossible to rouse her in the slightest degree; but, when fluid was put into her mouth, she swallowed without much difficulty, so that she took a full-sized wineglassful of brandy-and-water in the course of ten minutes.

The indications for treatment being obviously to sustain the heart's action until the effect of the chloral had passed off, we determined to give her brandy and diffusible stimulants, as far as possible, by the mouth, and to supplement our efforts in that direction by repeated injections up the bowel of strong beef-tea and brandy. However, everything proved unavailing. She continued in much the same condition until about nine o'clock the following morning, when she sank, without having exhibited the slightest consciousness, or moved a muscle, from the time she fell asleep on the evening of the 31st of December.

Judging from what I have learned from various members of our profession, I believe that, although fatal consequences may rarely follow a dose of thirty grains of the chloral hydrate, yet that unpleasant, if not dangerous, symptoms, are not unfrequently experienced. Dr. Tuke informs me that in a man whom he saw suffering from the effects of intemperance, thirty grains very nearly proved fatal, the symptoms of depression and failure of the heart's action being most alarming; and Mr. Fred. Webb, of Maida-vale, has given me the particulars of another case, in which an elderly man very nearly lost his life from the effects of thirty grains. The faintness, pallor, and depression of the heart's action, were excessive, and for some time Mr. Webb was in doubt whether he would be able to sustain the pulsations of the heart until the effects of the chloral had passed.

Doubtless these cases are quite exceptional, and are met with only in about the same proportion as cases of death from the administration of chloroform. But the facts I have cited are sufficient to prove that such cases are more frequent than is commonly supposed. Further, they inculcate the necessity for caution in the administration of the drug; and they point to the conclusion that thirty grains form too large a dose for ordinary use, and especially for a patient in whom the effects of the chloral have not been previously tried. As a hypnotic, in nervous restlessness, ten or fifteen grains ordinarily prove efficacious; and I have not seen or heard of any unpleasant symptoms following these doses. But the cases above recorded prove that larger doses, though usually harmless, and often marvellously efficacious, are not unattended with risk; and now that the public are beginning to dose themselves with chloral, as they have done of late years with chlorodyne, this important fact cannot be too widely known.—Manchester Square, W., March 2nd, 1871.—*Nashville Journal of Medicine and Surgery.*

